# Barriers to postpartum screening for type 2 diabetes: a qualitative study of women with previous gestational diabetes

**DOI:** 10.11604/pamj.2017.26.54.11433

**Published:** 2017-02-01

**Authors:** Forough Rafii, Seyedeh Fatemeh Vasegh Rahimparvar, Neda Mehrdad, Afsaneh Keramat

**Affiliations:** 1 Nursing Care Research Center, Nursing and Midwifery Faculty, Iran University of Medical Sciences, Tehran, Iran; 2 Nursing Care Research Center, Iran University of Medical Sciences, Tehran, Iran; 3 Nursing and Midwifery Faculty, Tehran University of Medical Sciences, Tehran, Iran; 4 Endocrinology and Metabolism Research Center, Endocrinology and Metabolism Clinical Sciences Institute, Tehran University of Medical Sciences, Tehran, Iran; 5 Nursing and Midwifery Faculty, Shahrood University of Medical Sciences, Shahrood, Iran

**Keywords:** Gestational diabetes mellitus, postpartum, qualitative research, screening, type II diabetes

## Abstract

**Introduction:**

Risk of developing type 2 diabetes is increased in women with previous gestational diabetes mellitus (GDM). Postpartum glycemic screening is recommended in women with recent GDM. But this screening rate is low and the reasons are unclear. The aim of this study was to explore the experiences of Iranian women with recent GDM on barriers of postpartum screening for diabetes.

**Methods:**

This qualitative study was conducted in Tehran, Iran in 2016. Semi-structured interview was used for data collection. 22 women with recent GDM were interviewed. These women gave birth in Tehran hospitals at a minimum of 6 months before interview. The missed screening defined as not attending to laboratory for Fasting Blood Sugar and/or Oral Glucose Tolerance Test, 6 week to 6 month after their child birthing. The data was analyzed by content analysis method.

**Results:**

Themes and sub-themes that illustrated the barriers to postpartum diabetes screening were: inadequate education (about developing diabetes in the future, implementation of the screening, and glucometer validity in diagnosis of diabetes), perceiving the screening as difficult (feeling comfortable with the glucometer, poor laboratory conditions, issues related to the baby/babies, and financial problems), improper attitudes toward the screening (unwilling to get diagnosed, not giving priority to oneself, having false beliefs) and procrastination (gap to intention and action, self-deception and self-regulation failure).

**Conclusion:**

Women with recent GDM reported several barriers for postpartum diabetes screening. This study help to develop the evidence-based interventions for improving this screening rate.

## Introduction

Gestational diabetes mellitus (GDM) defined as diabetes diagnosed during pregnancy. The prevalence of GDM is increasing [1]. In a systematic review the prevalence of GDM in Iranian pregnant women was 4.9% (CI 95%: 3.9-5.8) [2]. GDM may be related to beta cell defect and insulin resistance [3]. Therefore recognizing GDM allows recognition of women at increased risk of developing diabetes in future [4]. GDM usually resolves after delivery. But in studies that examined women with a history of GDM from 6 weeks to 28 years after delivery, the cumulative incidence of diabetes ranged from 2.6% to over 70%, also increased obviously in the first 5 years after delivery [5]. Women with recent GDM should be followed to classify their glucose status at least 6 weeks after delivery [6]. American Diabetes Association (2016) recommended an oral glucose tolerance test (OGTT) at 6 to 12 week postpartum for postpartum follow-up in women with GDM [7]. Identifying women at high-risk of developing diabetes may provide an opportunity to counsel with the woman about changing their lifestyle to prevent or delay the onset of diabetes [8, 9]. Despite the increased risk of developing type 2 diabetes in women with previous GDM, and recommendation for postpartum screening for diabetes, the attendance rate of the women for the screening is undesirable, which range from 18% to 57% [10–15]. The reasons for poor screening, especially in particular groups of women, are unclear [16, 17], and the majority of studies are from high-income countries [15]. Asian ethnicity is a known risk factor for development of GDM [15, 18]. Also Asian ethnicity is associated with higher postpartum glucose screening rates [11, 15]. Therefore, the Asian population is a good candidate to determine factors related to attendance to postpartum glucose screening, but most of the studies included Asian as a minor population [15]. In addition the available qualitative studies to explore the experiences of the women with previous GDM for understanding obstacles to the screening is rare [1, 16]. Also no studies is discovered in Iran, as an Asian country, about this context. So understanding Iranian women's experiences about obstacles of postpartum diabetes screening could provide the development of interventions to improve the attendance rate of the women to the screening. The aim of this study was to explore the experiences of Iranian women diagnosed with GDM on obstacles of postpartum screening for diabetes.

## Methods

**Design and Sampling:** This is a qualitative study conducted in 2016 in Tehran, Iran. We selected the women with previous GDM diagnosis by the hospital records of the women that delivered a minimum of 6 months before interview, by purposeful sampling method. We find the telephone number from the women's hospital records. During an initial phone call with these women, they were provided with a detailed description of the study and the informed consent procedures. The participants' demographic and clinical characters were collected during interview or their hospital records about their pregnancy and delivery. All interviews were conducted by the responding author at a place and time of the women's choice, usually in their home, and lasted for 25-50 minutes. A semi structured interview guide included open-ended questions were used. Women were asked about their general well-being, pregnancy and postpartum experiences. If they attended in postpartum diabetes screening, the questions were focused on the reasons they were able to attend and what helped or made it difficult to attend the screening. If they did not attend the screening, the further questions were about the reasons they missed the screening. We defined missed postpartum diabetes screening as not attending to laboratory for Fasting Blood Sugar or OGTT, from 6 week to 6 month after delivery. The interview guide was modified as the interviews progressed. So the subsequent questions were framed around the previous responses of the participants. For example, we incorporated more questions about measuring blood sugar by glucometer in postpartum after several participants spontaneously discussed these concepts. Also the interviewer used the reflective probes to encourage the women to further explain their perceptions. Also we used theoretical sampling focused to collect the most appropriate data to gain a deeper understanding of the women's experiences and perspectives on postpartum diabetes screening, with best variation and diverse backgrounds. For example after interviewing with the women who delivered in governmental hospitals, we interviewed with the women who delivered in private hospitals. We continued to sample until no new data emerged, based on thematic saturation.

**Data Analysis:** MAXQDA software 10 was used to facilitate data management and analysis. The data was analyzed by a content analysis method [19] and data trustworthiness was achieved using Lincoln and Guba´s Evaluative Criteria [20]. The data analysis was started after first interview. In order to analyze the data, all interviews were digitally audiotaped and transcribed verbatim, and the transcripts were re-read for several times, for facilitating identification of analytical categories. Then these were organized based on developing themes and subthemes. General themes and subthemes summarizing obstacles to postpartum diabetes screening were discussed among the co-authors, facilitating final interpretation of the data.

## Results

25 interviews with 22 women were conducted in this study. One participant decline to be interviewed because her husband did not want her to participate. The women's characteristics were summarized in Table 1. Four main themes were extracted as obstacles to postpartum diabetes screening. Table 2 presents the themes and sub-themes pertaining to these obstacles.

**Table 1:** Demographic and clinical characteristics of the participants

Participant No.	Age year	Educational level	Occupation	Diabetes in family	Woman’s healthy	Hospital of child birth	Prior GDM	Using insulin in pregnancy	Baby’s age, month	Baby’s healthy	Children no.	Breast-feeding	Screening
1	30	Primary	Housekeeper	Mother	Yes	Governmental	No	Yes	8	Yes	2	Yes	NO
2	33	University	Governmental job	Father	Yes	Private	No	Yes	6	Yes	1	Yes	Yes
3	24	University	Governmental job	No	Yes	Governmental	No	NO	11	No	1	Yes	NO
4	32	University	Governmental job	Mother	No	Governmental	No	Yes	17	No	2	Yes	Yes
5	31	University	Housekeeper	No	Yes	Governmental	No	NO	15	No	2	No	Yes
6	37	Illiterate	Illiterate	No	No	Governmental	Yes	Yes	12	No	2	NO	Yes
7	31	Diploma	Housekeeper	Mother	No	Governmental	No	NO	10	No	2	NO	Yes
8	32	High school	Self-employed	Father	Yes	Governmental	Yes	NO	7	Yes	2	Yes	No
9	31	Diploma	Housekeeper	No	Yes	Governmental	No	No	12	Yes	1	Yes	No
10	32	Diploma	Housekeeper	No	Yes	Private	No	Yes	14	Yes	2	Yes	Yes
11	40	Primary	Housekeeper	No	Yes	Governmental	No	Yes	18	Yes	1	Yes	Yes
12	42	Diploma	Self-employed	Mother, sister	Yes	Governmental	No	Yes	19	Yes	2	Yes	No
13	25	Primary	Housekeeper	No	Yes	Governmental	No	Yes	10	NO	1	Yes	No
14	32	Diploma	Housekeeper	Mother	No	Governmental	Yes	Yes	21	No	1	Yes	No
15	26	High school	Housekeeper	Mother, father	No	Governmental	No	No	8	Yes	1	Yes	Yes
16	31	Diploma	Housekeeper	No	Yes	Governmental	No	No	18	Yes	3	Yes	No
17	33	University	Governmental job	No	Yes	Private	No	Yes	8	Yes	2	Yes	No
18	31	Diploma	Governmental job	Mother	Yes	Private	No	Yes	7	Yes	2	Yes	No
19	25	High school	Housekeeper	Father	No	Governmental	No	Yes	8	Yes	1	No	Yes
20	34	Diploma	Governmental job	No	Yes	Governmental	No	Yes	9	Yes	3	Yes	Yes
21	33	University	Housekeeper	No	Yes	Governmental	No	No	7	Yes	2	Yes	No
22	31	Diploma	Housekeeper	Mother	Yes	Private	No	Yes	8	Yes	2	Yes	No

**Table 2:** Summary of the themes and the subthemes

Themes	Subthemes
**Inadequate education**	Developing diabetes in the future
	Implementation of the screening
	Glucometer validity in diagnosis of diabetes
**Perceiving the screening as difficult**	Feeling comfortable with the glucometer
	Poor laboratory conditions
	Issues related to the baby/babies
	Financial problems
**Improper attitudes toward the screening**	Unwilling to get diagnosed
	Not giving priority to oneself
	Having false beliefs
**Procrastination**	Gap to intention and action
	Self-deception
	Self-regulation failure

**Inadequate education:** According to our participant's perceptions, adequate education on the screening was not often given by healthcare staff to them. In addition most of them stated that they received the education only after delivery of their baby and not during their pregnancy. This theme consisted of three sub-themes. The first sub-theme was inadequate education about “developing diabetes in the future”. Most participants stated that they received no education on the likelihood of developing diabetes in the future due to their history of GDM. Sometimes, the women knew that they should visit a doctor for screening; however, they were not aware of the actual importance of the screening and thought that they had to undergo screening just because it was their doctor's recommendation. But most women said that they had been told that their diabetes will improve after delivery. In this regard participant No. 11 said: *“What the doctor says has an impact, you know? The doctor didn't tell me these things (that you may develop diabetes later on) … She only told me that my gestational diabetes will improve after the delivery”*. Some participants expressed that no education was given to them on the implementation of postpartum diabetes screening. The tertiary themes extracted from this subtheme included: education only if the women was getting insulin injections, failing to write the screening test, not advising to follow-up appointment, the lack of guidelines, and the non-mandatory nature of the screening education in the postpartum visits. According to the participants, postpartum diabetes screening was often not recommended to the women who did not get insulin injections and controlled their glucose levels only with their diet during pregnancy. In fact, the implementation of postpartum diabetes screening was advised only to women who received insulin injections during their pregnancy. Participant No. 3 that controlled her GDM only with her diet stated: *“There was another woman with me (after the delivery at the hospital). She said that she received insulin injections…They told her to get tested after the delivery…But they said no such thing to me”*. Failing to write the screening test was another tertiary theme. According to the participants' statements, sometimes there was not any written test for postpartum diabetes screening. Not advising to follow-up appointment was the other tertiary theme. In our study almost all women referred to Health Centers for vaccinating their child. But a few women referred for their own postpartum visits. Also most participants stated that when they referred to Health Centers, they were not recommended to undergo postpartum diabetes screening. Participant No. 3 said: *“At the Health Center that I visited, they advised me to take a pap smear only. They said nothing about diabetes and the test”*. The third subtheme was inadequate education about glucometer validity in diagnosis of diabetes. In our study the majority of the participants used home glucometers for controlling their blood sugar in pregnancy and measured it with glucometer at least one time in postpartum. Most participants claimed that the healthcare staffs provided all the information about how valuable glucometer values are for controlling blood sugar in pregnancy; however, the staffs did not inform them that glucometer does not provide a valid tool for diagnosis of diabetes after delivery. Participant No. 3 stated: *“Once or twice (after delivery), I decided to go to the lab to get tested, but the doctors said (during my pregnancy) that there was not a great discrepancy between the lab results and the home blood glucometer results, so I felt that everything was in check and did not go”.*

**Perceiving the screening as difficult:** Perceiving postpartum diabetes screening as difficult was noted to be another common obstacle. Feeling comfortable with the glucometer, poor laboratory conditions, issues related to the baby/babies, and financial problems were sub-themes extracted from this theme. Feeling comfortable with the glucometer was one of the most common cases. Most participants used home glucometers during pregnancy. They therefore still had access to glucometers after the delivery. Some women reported that they did not present to laboratory for the screening because they could easily measure their glucose levels using their home glucometer. In some cases, although the participants knew that the laboratory test was more precise and that the doctor had ordered the lab test, they did not present to a laboratory; instead, they measured their glucose levels with their home glucometer and interpreted the values themselves. Participant No. 1 expressed: *“At first (right after the delivery), I used to frequently measure my blood glucose whenever I ate, and since I noticed that my values were normal and did not go higher than 120…I did not go to the laboratory, even if the doctor had ordered so,…I opted for the easier option”.* Some participants suggested that poor laboratory conditions made postpartum diabetes screening all the more difficult. Some also noted the long time the test took, their long physical distance from the laboratory and the long queues in the laboratory as the others difficulties of the screening. Participant No. 18 stated: *“The test takes so long and not only do you have to be fasting once you take it, you should first drink a solution and then wait for two hours before you can get tested”*. As reported by most of the participants, issues related to the baby/babies comprised another obstacle to screening. Sometimes, breastfeeding during the first months after birth or problems following the child's illness construed obstacles to undergoing the screening, as they prioritized meeting their child's/children's needs over taking care of their own health. Also the difficulties of childcare, including having no one with whom to leave the baby/babies at home and difficulty of taking the baby to the laboratory were the problems faced by some mothers. In this regard, participant No. 1 said: *“It takes 10-15 minutes to go to the lab on foot, but it is difficult with a child…I have to wait there and the baby gets restless. Someone needs to take care of my baby while I'm gone”*. Also participant No. 12 stated: *“Because of my children, I cannot go out much…There is no one to keep an eye on them while I'm gone”*. Financial problems comprised another issue raised by some of the participating women, consisting of insurance-related problems such as the lack of insurance and expired insurance time. Participant No. 14 complained: *“Our insurance company doesn't cover this test right now. My insurance has expired… I had to pay 90,000 Tomans for just a couple of tests (with objection)”*. Not prioritizing the screening for financial reasons was another obstacle. Due to financial problems, some women preferred to spend their meagre income on their child/children or for other urgent costs that incurred rather than for undergoing the screening. Participant No. 12 stated: *“I don't really need (testing) … only because of how much it costs, since we are in a terrible financial position”*.

**Improper attitudes toward the screening:** Being unwilling to get diagnosed was construed an inappropriate attitude possessed by some participants. This women despite knowing that they were at higher risk for developing diabetes, they were unwilling to face the truth. To escape the truth about having diabetes, they preferred not to visit for screening. In this case participant No. 4 said: *“Sometimes you just don't want to believe that you're sick…I'm always afraid of learning that I'm sick”*. Not giving priority to oneself was another obstacle to screening. Some participants had problems which they preferred to get fixed first and then attend to their own health. Participant No. 14 expresses: *“I ignored my own health and decided not to go to the doctor's, because the other problems that I had were more important than my own health, so I said I'll handle these first”*. Having false beliefs was another obstacle to screening. Some women believed that they will not develop diabetes and therefore do not need screening because they breastfed, had a controlled diet and exercised. Some participants believed that since they showed no symptoms of diabetes, they did not need screening. Participant No. 8 stated: *“Since I comply well with my diet and also breastfeed my baby, I don't think my blood sugar can rise that high. I really comply with my diet”*.

**Procrastination:** Procrastination was another obstacle to the screening. Some participants stated that they think about undergoing screening, but took no action for it. Also they said that they were aware of the consequences of failing to undergo screening and were worried about it, but still overlooked it; so we extract Gap to intention and action subtheme. Participant No. 13 said: *“They ordered me a test after my delivery, but I haven't gone to take it yet (with a smile)…I'm thinking about it (the test), I'm suffering, but I still fail to do what I have to do. Truth be told, I'm always like this”*. In many cases, when the participants reported their reasons for the failure to present for screening, it looked as if they were merely self-deception. In other words, it looked that, despite the existing problems, these women could present for screening. Also the reasons they stated for their neglectful behavior appeared contrived, as they were seemingly only trying to deceive themselves or the researcher. According to the participants' statements it is suggested that a main reason for their failure to undergo screening seemed to be their extreme tendency to self-regulation failure. In this regard some of the women reported their shortage of time as the reason for not undergoing postpartum diabetes screening, while it seems that lack of time management was the main reason. Participant No. 14 stated: *“I had no time to go… Always I tell I do it tomorrow…but I do not gone again, because I have to do another duty …So I did not go to lab yet”. Also Participants No. 13 expresses:“I think I needlessly postpone going to lab…I habitually postpone going to lab until later…maybe with a good program I will be able to do it (go to lab)”*.

## Discussion

According to the experience of our participants, inadequate education was reported as a obstacle to postpartum diabetes screening. This is consistent with previous studies that have high-lighted inadequate education/information on diabetes screening after delivery [8, 21]. In our study, the screening was often not recommended to the participants who did not get insulin injections so these women did not attend to the screening. There was contradictory evidence about an association between insulin dependent GDM and diabetes post-partum screening [4, 8, 11–13, 22]. It seems that a main reason for controversy surrounding these findings is the difference in education to the women with insulin treatment and the women without insulin treatment by health care providers. The majority of our participants referred to Health Centers for getting their children vaccinated and a minority also presented for postpartum follow-up visit; however, postpartum diabetes screening education was often not provided in these centers to women with a history of GDM. As suggested by Seshiah (2015), mothers definitely attend the clinic for their baby immunization program and this opportunity can be utilized for postpartum screening [23]. Other studies showed that women attendance at the postpartum visit is a major positive factor in screening [11, 13]. Participation in screening can be improved through the encouragement of women with a history of GDM to present for postpartum follow-up care and through providing proper education, and similarly the mothers' attending for their baby's vaccination can be utilized. It is suggested that proper guidelines is developed and implementation of the screening is monitored by the authorities. Providers in Health Centers are recommended educate and encourage the women with a history of GDM for diabetes screening. Midwives are the main healthcare providers in contact with new mothers; it is suggested that they could a more effective role in education and encouraging the women for the screening.

Perceiving screening as difficult was another obstacle in our study. Capula et al. (2014) highlighted patient inconvenience as a most commonly factor associated with postpartum diabetes screening [21]. In our study almost all of the women had home glucometers and measured their glucose levels after their delivery, and some of them had not presented to the laboratory for the screening. Other studies also reported that measuring glucose levels with home glucometers was the reason that some women never presented to healthcare centers for proper screening [24]. Most of our study participants have measured their glucose levels several times after the delivery of their child, we suggested that they were given the device's ease of use. So we recommended to develop strategies for using the glucose levels shown by home glucometers in the postpartum diabetes screening session. The importance of this issue lies in the fact that if glucose values of glucometer that diagnose postpartum diabetes or pre-diabetes as postpartum diabetes screening will be determined, most women with a history of GDM will measure their postpartum glucose levels by home glucometers; so the rate of postpartum diabetes screening will be progress. Also in line with our findings, other studies have also reported laboratory-related problems [25], childcare [17] and higher parity [10, 13] as obstacles against screening. In our study, procrastination was an important factor that was associated with failure to postpartum diabetes screening. Based on the definition and other studies about procrastination, some subthemes was extracted: Gap to intention and action [26], self-deception [27, 28], self-regulation failure [29] and willingness to keep the daily routine. Procrastination is at the center of several societal problems, from the environment to our health [26]. Procrastination is shown as a vulnerability factor for poor adjustment to and management of hypertension and cardiovascular disease [30]. In the procrastination health model, procrastination is suggested to result in less frequent practice of health-promoting behaviors [30, 31]. Azami-Aghdash et al. (2015) reported that procrastination was as a key obstacle to screening of breast cancer [32]. In a follow-up survey, Keely et al. (2010) demonstrated that there is a neglected obstacle that affect postpartum diabetes screening rates, despite the perceived importance of screening by the patients [33]. According to our finding, we suggested that procrastination is the missed obstacle that affect postpartum diabetes screening in the patients of Keely's study. Nevertheless, it appears that in spite of all the problems and reasons proposed by the participants, the screening can be easily pursued with proper planning and with the avoidance of procrastination. Finally in our opinion, procrastination is an important missed factor for poor postpartum diabetes screening. About the limitations of this study, similar to other qualitative studies, the findings of our study were derived from perspective of women with a recent GDM who gave birth in hospitals of Tehran. So the transferability of the finding will be limited to comparable setting.

## Conclusion

Women with recent GDM reported several obstacles for postpartum diabetes screening, four main obstacle is included: inadequate education, perceiving the screening as difficult, improper attitudes toward the screening and procrastination. Based on our findings, the training of women with GDM should be taken more seriously in Iran during the women's pregnancy, delivery and postpartum period. Proper guidelines should be developed for hospitals and Health Centers and private clinics and its implementation should be monitored by the authorities. Healthcare providers are recommended to take advantage of the opportunity provided by new mothers' routine postpartum visits for both their own checkup and their child's vaccination and to train and encourage them for screening. Midwives are the main healthcare providers in contact with new mothers; they should therefore have a key role in training and encouraging women for screening during pregnancy, delivery and the postpartum period. Due to the availability of glucometers for women with a history of gestational diabetes, since most of these women have routinely used glucometers during their pregnancy, and given the device's ease of use and the fact that most of these women have measured their glucose levels several times after the delivery of their child, researchers are recommended to develop strategies for using the glucose levels shown by home glucometers in the postpartum diabetes screening session at the laboratory too. This issue is very pressing. It seems that if a range of glucose values of glucometer is determined that diagnose postpartum diabetes or pre-diabetes as postpartum diabetes screening, most women with a history of gestational diabetes will measure their postpartum glucose levels using home glucometers. So the rate of the screening will be very much increased. Also procrastination appears to play an important role in the failure to present for screening; further studies are recommended to be conducted for confirming this impact and for developing evidence-based operational strategies for overcoming the issues.

### What is known about this topic

- Risk of developing type 2 diabetes is increased following gestational diabetes;
- Women with recent GDM is recommended to attend postpartum diabetes screening;
- Rate of postpartum diabetes screening is low and the reasons are unclear.

### What this study adds

- There is not sufficient education about postpartum diabetes screening to Iranian women specially the women without using insulin during their pregnancy;
- Procrastination is an important and missed obstacle of postpartum diabetes screening;
- Measuring of blood glucose with glucometer is popular and easy for women with recent gestational diabetes, in postpartum.
